# Supplementary material for: Loop Dynamics Mediate Thermal Adaptation of Two Xylanases from Marine Bacteria
Source: Int J Mol Sci. 2025 Mar 30;26(7):3215. doi: 10.3390/ijms26073215 (PMC11989904; doi:10.3390/ijms26073215)

Figure S1 Validation of Structural Models Created by SWISS-MODEL.

SWISS-MODEL provided a Global Model Quality Estimate (GMQE) score, which rates overall model quality from 0 to 1 (higher values indicate better expected quality). The CaXyn10B model had a GMQE of 0.98, while the ZgXyn10A model had a GMQE of 0.96. Both models were validated using the ERRAT program. Results showed the ZgXyn10A model had an overall quality factor of 94.065, and the CaXyn10B model had an overall quality factor of 96.232.

Program: ERRAT2
File: ZgXyn10A-model_01.pdb
Chain#:A
Overall quality factor**: 94.065

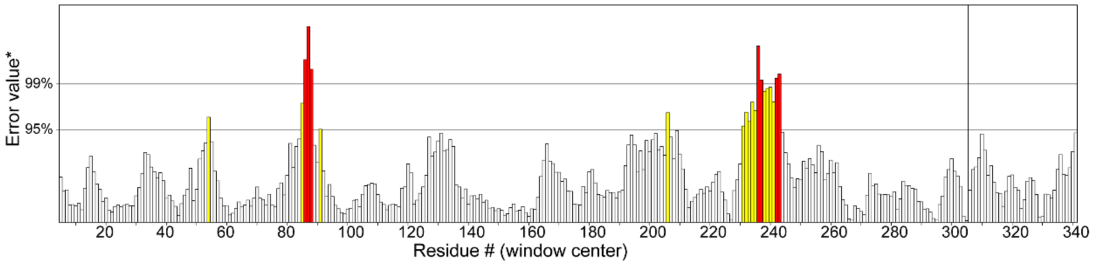

*On the error axis, two lines are drawn to indicate the confidence with which it is possible to reject regions that exceed that error value.
**Expressed as the percentage of the protein for which the calculated error value falls below the 95% rejection limit. Good high resolution structures generally produce values around 95% or higher. For lower resolutions (2.5 to 3A) the average overall quality factor is around 91%.

Program: ERRAT2
File: CaXyn10B-model_01.pdb
Chain#:A
Overall quality factor**: 96.232

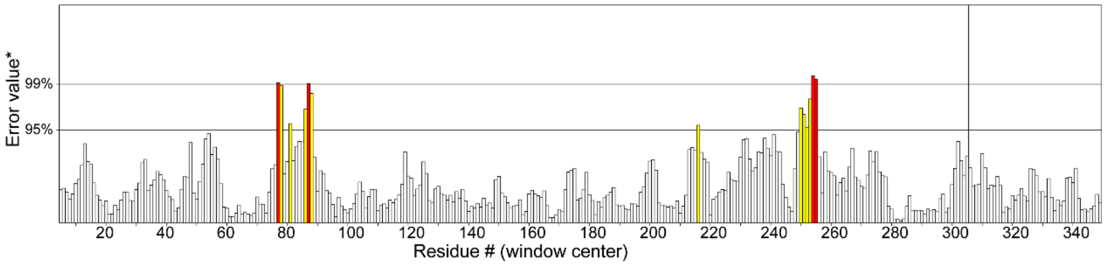

*On the error axis, two lines are drawn to indicate the confidence with which it is possible to reject regions that exceed that error value.
**Expressed as the percentage of the protein for which the calculated error value falls below the 95% rejection limit. Good high resolution structures generally produce values around 95% or higher. For lower resolutions (2.5 to 3A) the average overall quality factor is around 91%.

Figure S2. Michaelis-Menten curves for ZgXyn10A and CaXyn10B.

Enzymatic kinetics were measured using the DNS assay at 30°C and 40°C for CaXyn10B and ZgXyn10A, respectively. Substrate arabinoxylan concentrations ranged from 0.25 to 8 mg/mL. Michaelis-Menten curves were modeled via nonlinear regression, yielding R-squared values of 0.9909 for ZgXyn10A and 0.9918 for CaXyn10B.

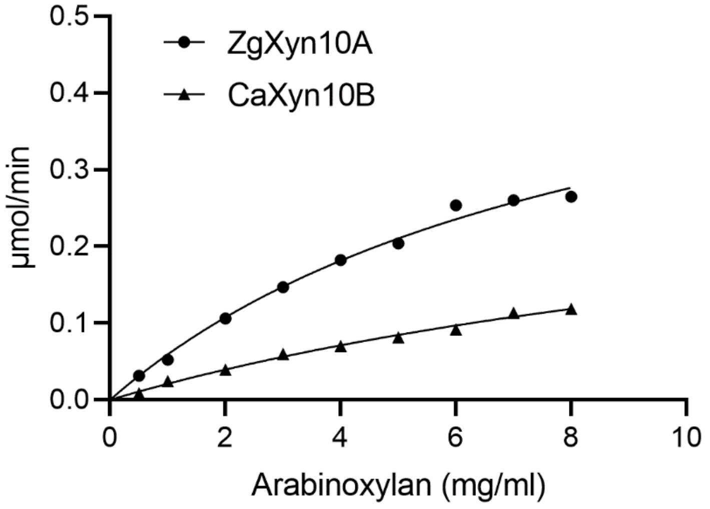

Figure S3. Temperature-dependent RMSD profiles for xylanases.

Molecular dynamics simulations were performed for ZgXyn10A, CaXyn10B, TmxB, and CoXyn10A at temperatures of 285K (blue), 300K (black), and 315K (red).

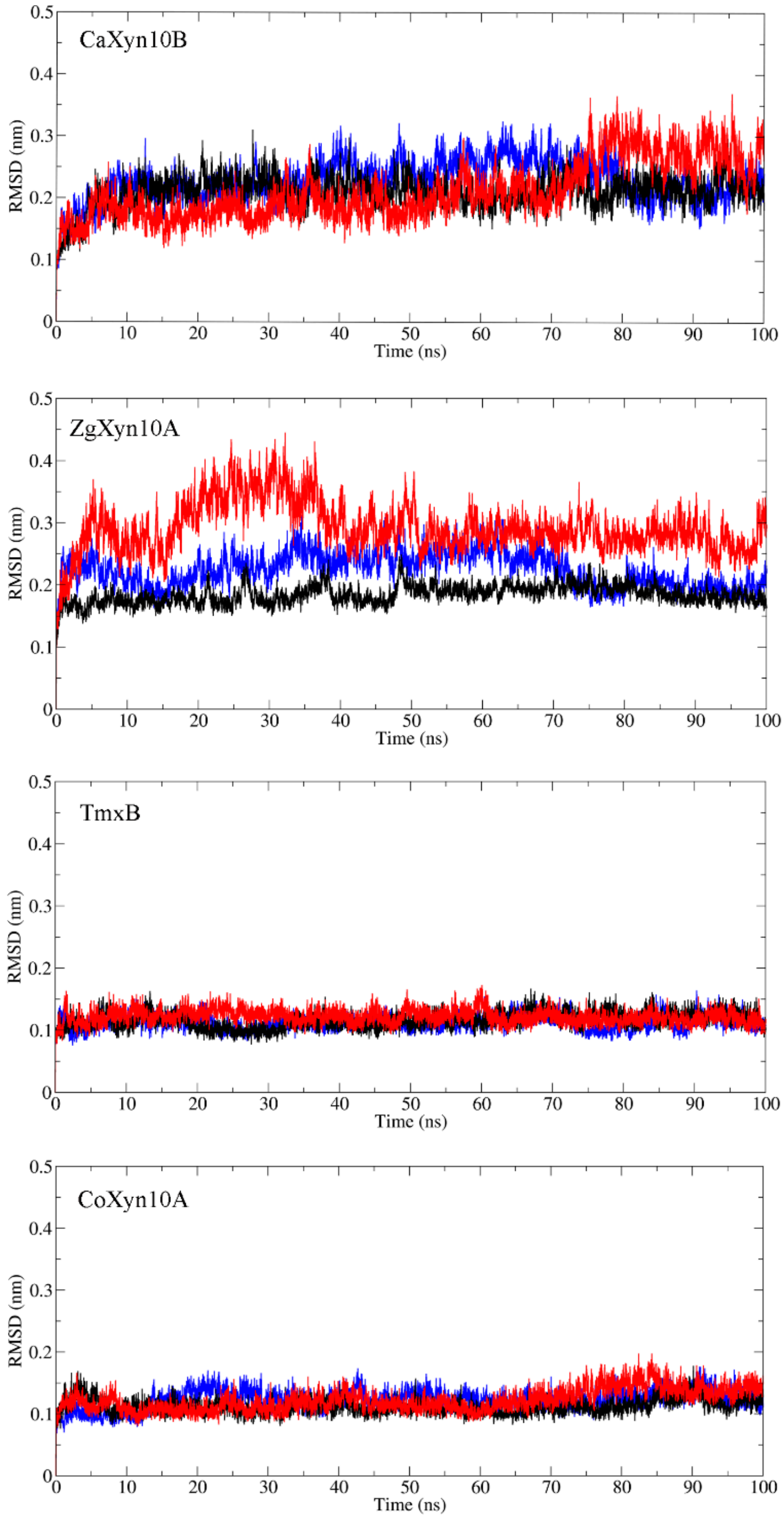

Figure S4. Temperature-dependent RMSF profiles for xylanases.

Molecular dynamics simulations were performed for ZgXyn10A, CaXyn10B, TmxB, and CoXyn10A at temperatures of 285K (blue), 300K (black), and 315K (red).

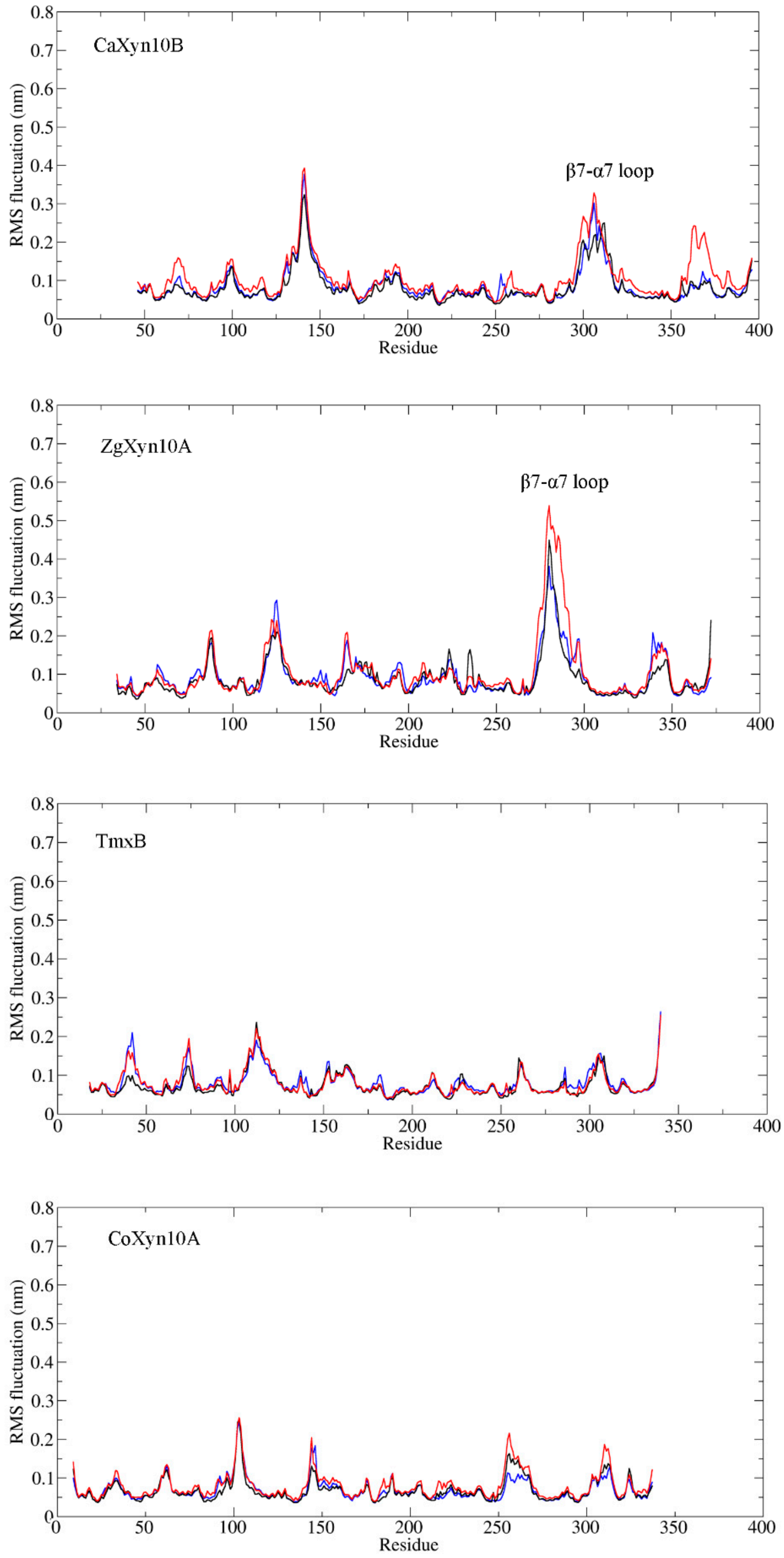

Figure S5. Hydrogen bond analysis for ZgXyn10A (green), CaXyn10B (blue), TmxB (red), and CoXyn10A (purple) at 300K.

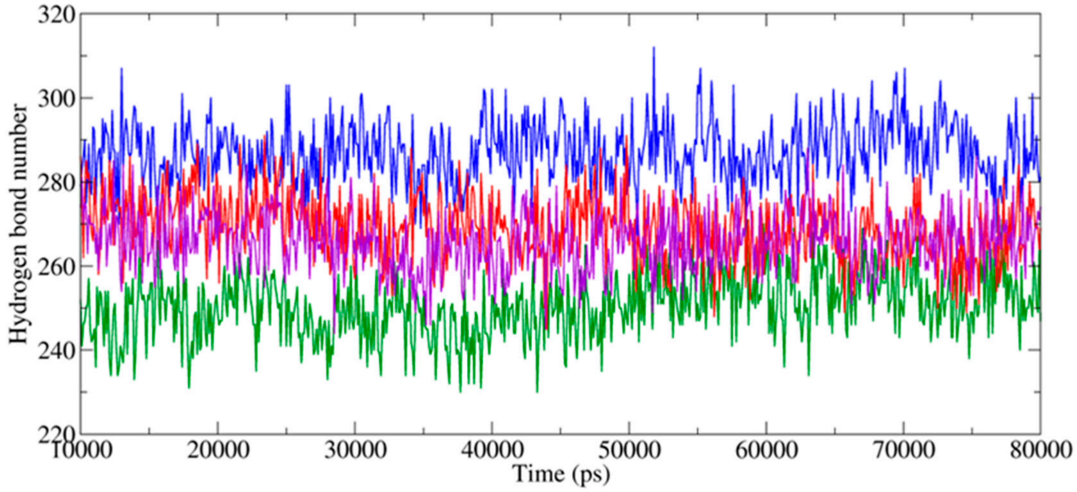

Supplement: Supplementary file 1 [file ijms-26-03215-s001.zip › ijms-3495956-supplementary.pdf]
